# Expression of the *siss-1* gene in *C. elegans*

**DOI:** 10.17912/micropub.biology.001976

**Published:** 2026-02-02

**Authors:** Marine Barsegyan, Cheryl Van Buskirk

**Affiliations:** 1 Biology, California State University, Northridge, Northridge, California, United States

## Abstract

*C. elegans* SISS-1 is an Epidermal Growth Factor (EGF) family ligand that signals from&nbsp;damaged cells to sleep-promoting neurons during s tress- i nduced s leep (SIS). Damage to a range of tissues can trigger SIS, and we reasoned that *siss-1* should be widely expressed. Here we investigate *siss-1* expression using both endogenous and transgenic fluorescent reporters. Our endogenous reporter reveals *siss-1* expression in the pharynx, gut, rectal gland, vulval muscles, and a subset of neurons. Our transgenic reporters reveal expression in additional cell types including the distal tip cell of the migrating gonad and vulF cells of the developing vulva. This expression is specific relative to our expectation and suggests that not all tissues are capable of signaling to sleep neurons.

**Figure 1. Expression of siss-1 as revealed by endogenous and transgenic fluorescent reporters:**
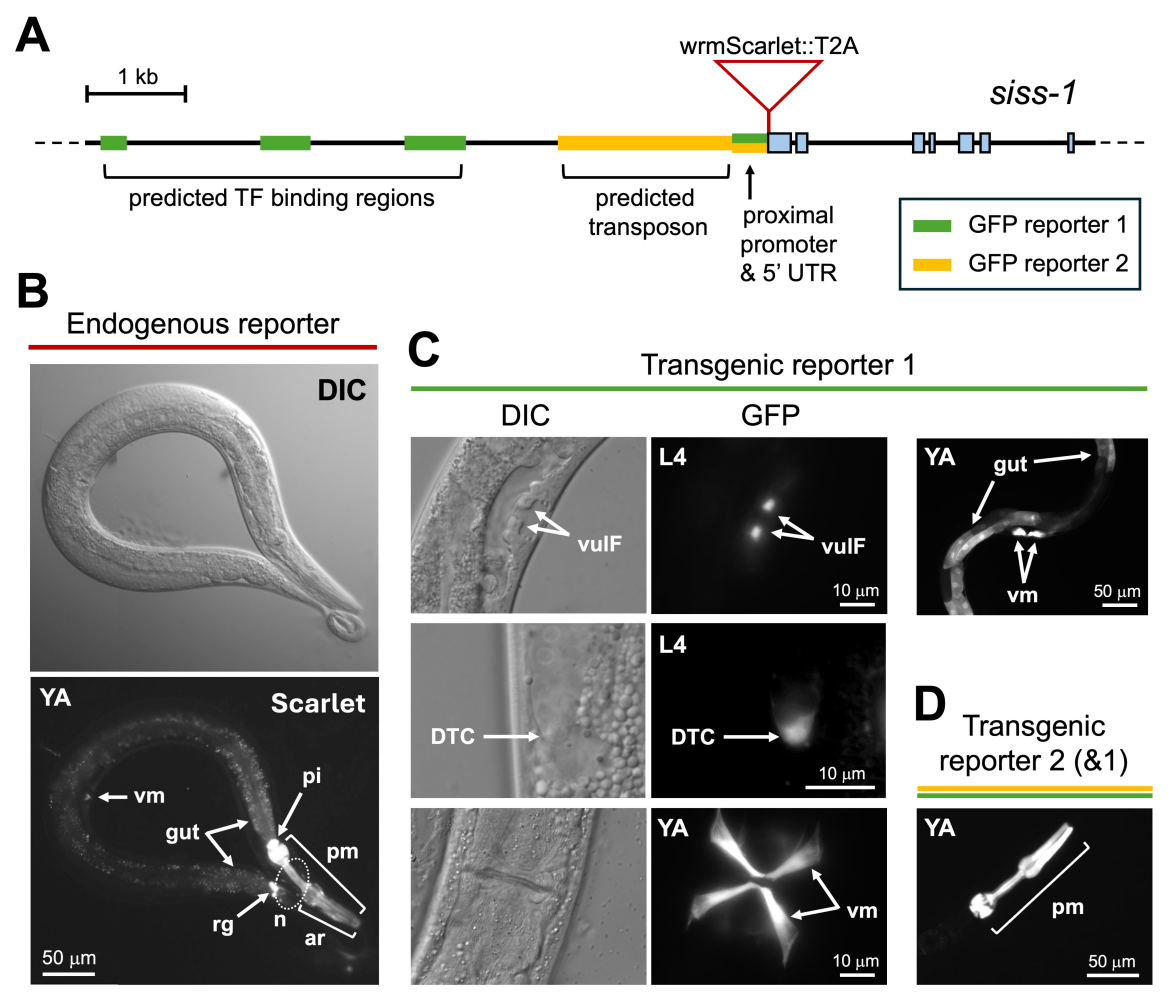
(A) Schematic of the *siss-1* gene and upstream region. An endogenous reporter (red) was created by knock-in of wrmScarlet and T2A peptide at the start codon of *siss-1* . Two transgenic GFP reporters were created using predicted transcription factor (TF) binding regions, a predicted transposable element, the proximal promoter and short 5'UTR. (B) Expression of the *siss-1* endogenous reporter, showing strong expression in pharyngeal muscle (pm), the pharyngeal-intestinal valve (pi) and rectal gland (rg), moderate expression in the arcade cells (ar), gut, and vulval muscle (vm), and weak expression in neuronal cell bodies (n) of the nerve ring and tail. (C) Transgenic GFP reporter 1 shows a similar pattern but with additional expression within vulF cells and the distal tip cell (DTC) of L4 animals. This reporter shows stronger expression relative to the endogenous reporter in vulval muscles and gut. (D) While both transgenic GFP reporters show strong expression in pharyngeal muscle, this is the only site of expression from reporter 2, shown here. L4 = fourth larval stage, YA = young adult stage.

## Description

*C. elegans* sleeps following exposure to a variety of damaging conditions, including ultraviolet (UV) light (DeBardeleben et al. 2017), wounding (Goetting et al. 2020), heat, toxin ingestion, and more (Hill et al. 2014; Nelson et al. 2014). This cellular stress-induced sleep (SIS) is mediated by activation of the Epidermal Growth Factor (EGF) receptor LET-23 /EGFR within sleep-promoting neurons (Hill et al. 2014; Konietzka et al. 2020). The EGF family ligand SISS-1 and the metalloprotease ADM-4 ,&nbsp; homologous to the stress-responsive sheddase ADAM17, are also required for SIS (Hill et al. 2024). Site of action studies point to a function for ADM-4 within damaged tissues (Hill et al. 2024), suggesting that SISS-1 is shed from damaged cells to promote EGFR activation within sleep neurons. For example, following exposure to the gut-specific toxin Cry5B, ADM-4 is required specifically within the intestine for the sleep response. As damage to a variety of tissues can trigger sleep, and in some cases sleep sets in within minutes of exposure, we reasoned that *siss-1* is likely to be widely and constitutively expressed, like *adm-4* (Ho et al. 2022). Here we investigate *siss-1* gene expression using both endogenous and transgenic fluorescent reporters ( Fig. 1A ).

We created an endogenous reporter via knock-in of wrmScarlet at the N-terminus of SISS-1 , with an intervening T2A peptide to allow independent translation of the fluorophore and SISS-1 (Ahier & Jarriault 2014). We observed strong wrmScarlet expression in pharyngeal muscle, the pharyngeal-intestinal valve, and the rectal gland ( Fig. 1B ). We observed moderate expression in the pharyngeal arcade cells, vulval muscles, and cells at the anterior and posterior ends of the intestine ( Fig. 1B ). Last, we observed weak expression in the nerve ring and in unidentified tail neurons ( Fig. 1B ). We did not detect fluorescence in the germline, body muscle, nor epidermis. The expression of *siss-1* did not appear to change following exposure to stressors (heat, UV, Cry5B toxin) and was consistent across developmental stages and sexes, apart from vulval muscles that arise only in hermaphrodite adults. The expression pattern of *siss-1* overlaps with but is more restricted than that of *adm-4* , which includes the pharynx, intestine, vulva, nervous system, gonad, and epidermis (Ho et al. 2022). As endogenous reporters are typically weaker than high-copy transgenic reporters, such as the *adm-4 p* :GFP reporter, it is possible that some *siss-1* expression is below our detection level. We therefore turned to transgenic reporter analysis.

We searched the 10 kb intergenic region upstream of *siss-1* for potential regulatory regions using the JBrowse feature of WormBase (Sternberg et al. 2024) and identified three predicted transcription factor binding regions (Gerstein et al. 2010). A synthetic DNA fragment combining these regions with the *siss-1* proximal promoter was fused to GFP ( Fig. 1A, GFP reporter 1), and multiple transgenic lines were established. This reporter showed expression in pharyngeal muscle, intestine, rectal gland, and vulval muscle, similar to the endogenous reporter, and revealed expression in vulF cells of the developing vulva and in the distal tip cell of the migrating gonad in L4 animals ( Fig. 1C ).

Last, we were interested in determining if a 1.8 kb truncated RTE-1 retrotransposable element just upstream of *siss-1* ( Fig. 1A ) might contain regulatory elements, and thus we examined a GFP reporter containing the proximal 2.15 kb of the *siss-1* upstream region. Transgenic animals carrying this reporter showed GFP expression exclusively in pharyngeal muscle ( Fig. 1D ). As both of our GFP reporter constructs show pharyngeal expression and have in common the 350 bp proximal promoter, we conclude that this proximal region drives expression in pharyngeal muscle, and that the retrotransposon lacks enhancer elements.

Our findings are consistent with transcriptomic data (Taylor et al. 2021; www.cengen.org) showing *siss-1* expression across stages in the rectal gland, pharyngeal muscle, arcade cells, intestine and neurons, with L4 expression in distal tip cells and L4/adult expression in vulval muscle. We expected to observe epidermal *siss-1* expression, as both the pharynx and epidermis appear to be sites of action for ADM-4 during UV-SIS (Hill et al. 2024). However, while *siss-1* expression in the epidermis is detected by transcriptomics, we did not detect epidermal expression with our reporters. It is possible that regulatory elements are missing from our transgenic reporters, and that epidermal expression is below the level of detection by the low-copy endogenous reporter. Interestingly, *siss-1* is most highly expressed in tissues of the alimentary system (pharynx, intestine, rectal gland), suggesting that cells exposed to ingested toxins and pathogens are major contributors to stress-induced sleep. Last, while the only known function of SISS-1 to date is in stress-induced sleep, its transient expression in certain cell types, such as the vulF cells and distal tip cells of the L4 larva, may point to developmental roles.

## Methods

**Endogenous *siss-1* reporter** : The *siss-1* reporter strain PHX9490 (SunyBiotech) harbors a CRISPR-mediated knock-in of wrmScarlet (El Mouridi et al. 2017), and a T2A peptide (Luke et al. 2008) at the start codon of *siss-1* (chr. IV:4562665). T2A is a ‘ribosomal skip' 2A peptide from *Thosea asigna* &nbsp;virus that allows independent translation of the fluorophore and the flanking protein in multiple systems including *C. elegans* (Ahier & Jarriault 2014).

**Identification of potential *siss-1* regulatory elements** : The JBrowse feature of WormBase was used to survey transcription factor binding regions having experimental evidence of binding sites for one or more transcription factors (Gerstein et al. 2010). The 10 kb upstream intergenic region of&nbsp; *siss-1* &nbsp;( F28E10.1 ) contains several such regions, and we chose to examine the three most supported by experimental evidence: a 270 bp region (4556131-4556400) 6.25 kb upstream of *siss-1* , a 500 bp region (4557700-4558200) 4.5 kb upstream of *siss-1* , and a 600 bp region (4559101-4559700) 3.0 kb upstream of&nbsp; *siss-1* . Among these regions, transcription factors predicted to bind include CEH-9 , DAF-16 , ELT-3 , FOS-1 , GMEB-1 , HDA-1 , HPL-2 , LIN-35 , NHR-6 , NHR-28 , NHR-77 , PHA-4 , PQM-1 , SKN-1 and UNC-62 . We also identified a 1.8 kb (4560500-4562290) partial RTE-1 retrotransposon sequence 350 bp upstream of&nbsp; *siss-1* .

**Composite regulatory region (GFP reporter 1):** &nbsp;&nbsp;A DNA fragment containing the three juxtaposed transcription factor binding regions (270, 500, and 600 bp) and the 350 bp proximal promoter was synthesized by Twist Biosciences. A BamHI site was introduced just 5' of the proximal promoter, and the entire fragment was flanked by 5' HindIII and 3' SmaI sites and cloned into the GFP expression vector pPD95.75 (Addgene plasmid #1494). Diagnostic digests revealed that the fragment had inserted at just the SmaI site, but in the intended orientation, resulting in pMB1. This construct was injected into N2 animals at 110 ng/ul by InVivo Biosystems. Animals were immobilized in 1 mM levamisole in M9 and imaged on a Zeiss Axio Imager A2. Three lines were examined, and a representative line is shown here.

**Proximal regulatory region (GFP reporter 2):** A DNA fragment corresponding to the 1.8 kb retrotransposon sequence was synthesized by Twist Biosciences flanked by 5' BamHI sites, cloned into pMB1, and verified for insert orientation. This replaced the three predicted regulatory regions of pMB1 with the transposon sequence and retained the proximal promoter, producing pMB2. This was injected into N2 animals at 110 ng/ul by InVivo Biosystems. Animals were immobilized in 1 mM levamisole in M9 and imaged on a Zeiss Axio Imager A2. Three lines were examined, and a representative line is shown here.

**Examination of *siss-1* expression across stages and conditions** :&nbsp; The endogenous and transgenic *siss-1* reporters were examined in adult hermaphrodites and males, as well as in each larval stage in hermaphrodites, with consistent expression patterns observed except for those noted in the text. At least 30 animals were imaged per stage. For examination of potential stress-responsive changes in *siss-1* expression, young adult animals were exposed to either UV-B light, Cry5B toxin, or 35˚C heat shock as previously described for triggering stress-induced sleep (Hill et al. 2024) and then imaged at several time points up to 3 hr after exposure. At least 30 animals were imaged per stress condition, with no discernible impact on *siss-1* expression.

## Reagents

**Table:** 

**Strain**	**Genotype**
PHX9490	*siss-1 ( syb9490* [wrmScarlet::T2A:: *siss-1* ])
CVB94	*siss-1* p::GFP reporter 1 (pMB1)
CVB92	*siss-1* p::GFP reporter 2 (pMB2)

All strains are available from the Van Buskirk lab upon request.
